# Development and validation of an explainable machine learning model for predicting interstitial fibrosis and tubular atrophy in biopsy-confirmed diabetic nephropathy

**DOI:** 10.3389/fendo.2026.1852512

**Published:** 2026-05-01

**Authors:** Huifang Wang, Longfei Li, Xiaodan Zhang, Demin Xie, Xia Li

**Affiliations:** 1 Department of Nephrology, The Affiliated Hospital of Qingdao University, Qingdao, China; 2 Medical Affairs Department, The Affiliated Hospital of Qingdao University, Qingdao, China; 3 Department of Critical Care Medicine, The Affiliated Hospital of Qingdao University, Qingdao, China

**Keywords:** diabetic nephropathy, interstitial fibrosis, machine learning, SHAP, tubular atrophy

## Abstract

**Background:**

Interstitial fibrosis and tubular atrophy (IFTA) are key pathological features of chronic kidney damage and progression in diabetic nephropathy (DN). Early identification of patients at higher risk of IFTA may support risk stratification, although reliable non-invasive tools remain limited. This study aimed to develop and validate machine learning (ML) models for predicting IFTA in patients with biopsy-confirmed DN.

**Methods:**

In this retrospective study, 232 patients with biopsy-confirmed DN from 2017 to 2025 were included and randomly divided into a training cohort (*n* = 164) and a validation cohort (*n* = 68). Baseline clinical and laboratory variables were collected. Feature selection was performed using least absolute shrinkage and selection operator (LASSO) regression with 10-fold cross-validation. Seven ML algorithms—logistic regression, support vector machine, random forest, XGBoost, LightGBM, decision tree, and artificial neural network—were developed. Model performance was evaluated using receiver operating characteristic curves, calibration plots, and decision curve analysis. Model interpretability was assessed using SHAP.

**Results:**

Seven predictors were identified, including diabetic retinopathy, age, proteinuria, estimated glomerular filtration rate (eGFR), triglycerides, duration of diabetes, and hemoglobin. Among the models, XGBoost achieved the highest AUC in the validation cohort, with an area under the curve (AUC) of 0.759, accuracy of 72.1%, sensitivity of 92.3%, specificity of 44.8%, and F1 score of 79.1%. Overall, the model showed moderate discrimination, with high sensitivity but limited specificity, suggesting potential value for exploratory risk screening rather than definitive clinical use. SHAP analysis indicated that higher proteinuria, triglycerides, presence of diabetic retinopathy, and longer diabetes duration, together with lower eGFR, hemoglobin, and younger age, were associated with an increased predicted risk of IFTA.

**Conclusion:**

ML models, particularly XGBoost, showed moderate performance in predicting IFTA in patients with biopsy-confirmed DN using routinely available clinical variables. These findings support the feasibility of an interpretable, non-invasive approach for exploratory risk estimation of tubulointerstitial injury. However, because of the modest sample size, limited specificity, relatively high false positive rate, and lack of external validation, the present results should be considered preliminary and require further validation before clinical use.

## 1 Introduction

Diabetic nephropathy (DN) is a common and progressive complication of type 2 diabetes and remains a major cause of chronic kidney disease and end-stage renal disease worldwide. Despite advances in glycemic control and renoprotective therapies, the clinical course of DN remains highly heterogeneous, highlighting the limitations of conventional clinical indicators for early risk assessment and the need for earlier risk stratification and more integrated approaches to disease assessment (1, 2). In addition, emerging evidence suggests that “medicine food homology” plants may exert therapeutic effects through modulation of metabolic pathways, oxidative stress, and inflammatory responses, thereby providing a complementary perspective on disease management and underlying pathological mechanisms (3).

Among the various pathological changes in DN, tubulointerstitial injury, especially interstitial fibrosis and tubular atrophy (IFTA), is a critical determinant of long-term renal outcome (4). The extent of IFTA has been shown to correlate more closely with renal function decline than glomerular lesions alone. However, in clinical practice, IFTA is typically assessed through renal biopsy, an invasive procedure not suitable for routine or repeated evaluation. Consequently, clinicians often lack reliable non-invasive tools to identify patients at high risk of significant tubulointerstitial damage before irreversible injury occurs.

Traditional markers, such as proteinuria and estimated glomerular filtration rate (eGFR), provide essential information but are insufficient to fully assess underlying renal damage. Patients with similar levels of proteinuria or eGFR may have markedly different degrees of IFTA, complicating early risk stratification and timely intervention (5). Recent advances in machine learning (ML) offer promising solutions to this challenge by integrating complex, multidimensional clinical data to identify non-linear relationships that traditional statistical methods cannot capture (6, 7). ML models, leveraging routinely available clinical variables, can provide individualized risk estimates reflecting underlying pathological processes, even in the absence of direct tissue assessment. Furthermore, the increasing emphasis on model interpretability allows clinicians to better understand how specific features influence predictions, thus improving clinical applicability and trust in these models (8).

In this context, we conducted a retrospective study to develop and validate machine learning-based models for predicting IFTA in patients with biopsy-confirmed DN. Unlike many previous studies that focused on general renal risk prediction, our study specifically aimed to estimate biopsy-defined tubulointerstitial injury using routinely available clinical data. We compared several machine learning algorithms and incorporated explainability methods to better understand how individual variables contributed to model predictions. In this way, we sought to provide an interpretable and non-invasive, but still exploratory, framework for pathology-oriented risk stratification in DN.

## 2 Materials and methods

### 2.1 Study population and design

This retrospective observational study was based on adult patients with type 2 diabetes mellitus who underwent renal biopsy at the Affiliated Hospital of Qingdao University between 1st January 2017 and 1st May 2025. Only patients with histopathologically confirmed diabetic nephropathy were considered eligible for inclusion. The primary objective of the study was to construct predictive models for the presence of IFTA using routinely available clinical information.

Patients were included if they were aged 18 years or older, had a confirmed diagnosis of type 2 diabetes mellitus, and demonstrated pathological features consistent with diabetic nephropathy on renal biopsy. Patients were excluded if biopsy findings indicated the coexistence of other primary or secondary kidney diseases, if essential clinical or laboratory data were missing, or if severe systemic illness or active infection was present at the time of biopsy.

The study protocol conformed to the principles of the Declaration of Helsinki and was approved by the Ethics Committee of the Affiliated Hospital of Qingdao University (approval number: QYFY WZLL 80565). As this study involved anonymized retrospective data, the requirement for informed consent was waived.

### 2.2 Clinical and pathological data acquisition

Clinical data were extracted from electronic medical records and represented baseline information obtained at the time of renal biopsy. Collected variables included demographic characteristics, medical history, blood pressure measurements, and laboratory indices. Laboratory parameters comprised hematologic indices, biochemical markers, and renal function-related variables. Blood samples were obtained after an overnight fast and analyzed using standardized automated platforms in the hospital’s central laboratory.

Renal biopsy specimens were processed according to institutional protocols. Tissue samples were examined using light microscopy with multiple staining techniques, including hematoxylin and eosin, periodic acid-silver methenamine, and Masson trichrome staining. Immunofluorescence analysis was performed on frozen sections using antibodies targeting immunoglobulins and complement components. Ultrastructural evaluation was conducted using electron microscopy when adequate tissue was available. All pathological assessments were independently reviewed by two experienced renal pathologists, with discrepancies resolved by consensus.

Interstitial fibrosis and tubular atrophy were assessed semiquantitatively. IFTA was defined as involving more than 25% of the cortical area, consistent with commonly accepted pathological criteria, and was treated as a binary outcome variable for model development (9).

### 2.3 Variable definitions

Diabetic retinopathy was diagnosed by ophthalmologists based on characteristic fundoscopic findings. Hypertension was defined as a systolic blood pressure of ≥140 mmHg and/or diastolic blood pressure of ≥90 mmHg, measured on multiple occasions, or the current use of antihypertensive medication. Mean arterial pressure was calculated as diastolic blood pressure plus one-third of the pulse pressure.

Renal function was estimated using the Chronic Kidney Disease Epidemiology Collaboration (CKD-EPI) equation. Proteinuria was quantified using 24-hour urinary protein excretion. All variables were defined prior to model construction and treated according to their original measurement scales unless otherwise specified during preprocessing.

### 2.4 Data preprocessing and feature selection

To assess model generalizability, the cohort was randomly divided into a training set (70%) and a validation set (30%). The data split was performed before feature selection and model development. Feature selection was conducted exclusively within the training set to reduce the risk of information leakage, and the selected variables were then carried forward for model construction and evaluation.

Feature dimensionality reduction was performed using Least Absolute Shrinkage and Selection Operator (LASSO) regression, which enables simultaneous variable selection and regularization, minimizing multicollinearity and the risk of overfitting. A 10-fold cross-validation procedure was used to determine the optimal regularization parameter. Variables with non-zero coefficients at the optimal penalty level were retained as predictors for subsequent model development.

### 2.5 Machine learning model development and evaluation

Using the selected predictors, multiple machine learning algorithms were trained to predict the presence of IFTA, including logistic regression, support vector machine, decision tree, random forest, extreme gradient boosting (XGBoost), light gradient boosting machine, and artificial neural network models. Model hyperparameters were optimized by grid search with 5-fold cross-validation within the training dataset, and additional tuning was performed to further explore the trade-off between sensitivity and specificity (10).

Model performance was evaluated in both the training and validation datasets, with the main results reported for the validation dataset. Discriminative ability was quantified using the area under the receiver operating characteristic curve. Calibration was evaluated through calibration plots and Brier scores. Clinical utility was examined using decision curve analysis. In addition, confusion matrices were used to derive accuracy, sensitivity, specificity, precision, and F1 score, providing a comprehensive assessment of predictive performance.

### 2.6 Model interpretability analysis

To enhance transparency and facilitate clinical interpretation, feature contributions within the optimal model were analyzed using Shapley Additive Explanations (SHAP). SHAP values were used to quantify the direction and magnitude of each variable’s contribution to individual predictions as well as overall model output (11). Features with larger absolute SHAP values were considered to have greater influence on prediction results.

These complementary approaches enabled both global and local interpretation of model behavior, bridging the gap between predictive performance and clinical interpretability.

### 2.7 Statistical analysis

Continuous variables were summarized as mean ± standard deviation or median with interquartile range, depending on distribution characteristics, while categorical variables were presented as counts and percentages. Group comparisons were conducted using appropriate parametric or non-parametric tests. Variables with excessive missingness were excluded, and remaining missing values were handled using multiple imputation techniques (12).

All statistical analyzes and model development procedures were performed using SPSS version 27.0, R version 4.5.1, and Python version 3.10. A two-sided P value of less than 0.05 was considered statistically significant.

## 3 Results

### 3.1 Patient characteristics

A total of 244 eligible individuals diagnosed with DN underwent biopsy from 1st January 2017 to 1st May 2025 at the Affiliated Hospital of Qingdao University. Among them, 12 individuals were excluded from analysis according to exclusion criteria. Eventually, a total of 232 patients with DN at the Affiliated Hospital of Qingdao University were included in this study. The included patients were divided into the training set (164 patients) and the validation set (68 patients) in accordance with a ratio of 7:3, with generally comparable baseline characteristics between the two cohorts. In the training and validation sets, the median age was 54.50 (45.00, 61.25) and 53.00 (45.00, 60.25) years, and 56 (34.15%) and 26 (38.24%) patients were females, respectively. The presence of IFTA was 57.32% and 57.35% in the training and validation sets, respectively (*p* > 0.05). Detailed baseline characteristics are summarized in **Table 1** and were generally comparable between the training and validation cohorts.

**Table 1:** Training set and validation set variability analysis.

Variables	Total (*n*=232)	Training set (*n*=164)	Validation set (*n*=68)	*P* value
Age, years (median, IQR)	53.50 (45.00, 61.00)	54.50 (45.00, 61.25)	53.00 (45.00, 60.25)	0.798
Female, n (%)	82 (35.34)	56 (34.15)	26 (38.24)	0.553
Body mass index, kg/m^2^ (mean ± SD)	26.16 ± 3.81	26.10 ± 3.76	26.30 ± 3.95	0.717
Systolic BP, mmHg (mean ± SD)	153.44 ± 21.70	153.98 ± 21.07	152.12 ± 23.25	0.553
Diastolic BP, mmHg (mean ± SD)	87.16 ± 13.13	87.93 ± 13.27	85.32 ± 12.67	0.170
MAP, mmHg (mean ± SD)	109.25 ± 14.40	109.95 ± 14.43	107.59 ± 14.28	0.257
Hypertension, n (%)	192 (82.76)	137 (83.54)	55 (80.88)	0.626
Coronary heart disease, n (%)	10 (4.31)	8 (4.88)	2 (2.94)	0.727
Diabetic retinopathy, n (%)	133 (57.33)	90 (54.88)	43 (63.24)	0.241
Duration of diabetes, years (median, IQR)	8.00 (4.00, 11.00)	8.00 (4.00, 10.00)	8.00 (4.00, 15.00)	0.570
Laboratory				
White blood cell count, 10^9^/L (median, IQR)	6.48 (5.40, 8.18)	6.53 (5.34, 8.20)	6.43 (5.50, 7.99)	0.964
Hemoglobin, g/L (mean ± SD)	113.81 ± 22.68	113.85 ± 22.08	113.69 ± 24.22	0.961
Platelet counts, 10^9^/L (median, IQR)	232.00 (194.00, 280.25)	233.00 (192.75, 281.00)	232.00 (201.50, 276.50)	0.602
Serum uric acid, μmol/L (median, IQR)	371.50 (329.00, 445.00)	370.00 (325.00, 445.25)	383.50 (336.50, 443.50)	0.453
Serum creatinine, μmol/L (median, IQR)	115.00 (83.60, 166.48)	114.50 (83.75, 163.75)	117.95 (83.10, 172.28)	0.908
eGFR, ml/min/1.73m^2^ (median, IQR)	44.84 (29.71, 70.17)	45.62 (30.75, 68.44)	44.29 (28.89, 73.58)	0.923
Serum albumin, g/L (mean ± SD)	30.41 ± 7.43	29.87 ± 7.53	31.71 ± 7.07	0.087
Triglycerides, mmol/L (median, IQR)	1.98 (1.33, 2.93)	2.01 (1.33, 3.05)	1.88 (1.33, 2.74)	0.500
Total cholesterol, mmol/L (median, IQR)	5.74 (4.45, 7.62)	5.85 (4.49, 7.52)	5.46 (4.42, 7.66)	0.596
LDL-C, mmol/L (median, IQR)	3.34 (2.42, 4.84)	3.38 (2.42, 4.84)	3.23 (2.46, 4.80)	0.982
HbA1c, % (median, IQR)	7.20 (6.40, 8.60)	7.20 (6.30, 8.50)	7.35 (6.60, 8.80)	0.073
Immunoglobulin G, g/L (median, IQR)	7.63 (6.17, 10.70)	7.34 (6.08, 10.30)	8.25 (6.40, 11.53)	0.109
Fasting blood glucose, mmol/L (median, IQR)	6.30 (4.97, 8.05)	6.15 (4.95, 7.88)	6.71 (5.10, 8.22)	0.537
Hematuria, n (%)	167 (71.98)	114 (69.51)	53 (77.94)	0.193
Proteinuria (g/day) (median, IQR)	5.27 (2.68, 7.48)	5.50 (2.92, 7.32)	4.65 (2.43, 8.11)	0.498
IFTA, n (%)	133 (57.33)	94 (57.32)	39 (57.35)	0.996
Arteriolar hyalinosis, n (%)	218 (93.97)	154 (93.90)	64 (94.12)	0.950
Oral antidiabetic drugs, n (%)	202 (87.07)	141 (85.98)	61 (89.71)	0.441
Insulin, n (%)	69 (29.74)	45 (27.44)	24 (35.29)	0.234
RAAS inhibitors, n (%)	151 (65.09)	108 (65.85)	43 (63.24)	0.703
SGLT2 inhibitors, n (%)	80 (34.48)	56 (34.15)	24 (35.29)	0.867

Data presented as mean ± standard deviation (SD) or median (IQR) or percentage.

eGFR, estimated glomerular filtration rate; IFTA, interstitial fibrosis and tubular atrophy; LDL-C, low-density lipoprotein cholesterol; MAP, mean arterial pressure; HbA1c, glycated hemoglobin A1c.

Of the 164 patients in the training set, 94 (57.32%) patients were classified into the IFTA group and 70 (42.68%) patients into the non-IFTA group. The baseline demographic, clinical, and treatment characteristics of patients with and without IFTA in the training cohort are shown in **Table 2**. Hypertension, diabetic retinopathy, duration of diabetes, white blood cell count, serum creatinine, proteinuria, insulin use, and SGLT2 inhibitor use were significantly more frequent or higher in patients with IFTA than in those without IFTA (*p* < 0.05). Hemoglobin and eGFR were significantly lower in patients with IFTA than in those without IFTA (*p* < 0.05). No significant differences were observed for the other variables listed in **Table 2** between patients with and without IFTA (*p* > 0.05).

**Table 2:** Clinical characteristics of DN patients at the time of kidney biopsy with or without interstitial fibrosis and tubular atrophy.

Variables	Total (*n*=164)	Any IFTA (*n*=94)	No IFTA (*n*=70)	*P* value
Age, years (mean ± SD)	52.90 ± 10.68	51.71 ± 10.85	54.49 ± 10.32	0.100
Female, n (%)	56 (34.15)	31 (32.98)	25 (35.71)	0.715
Body mass index, kg/m^2^ (median, IQR)	25.99 (23.80, 28.38)	26.15 (23.81, 28.95)	25.83 (23.80, 27.86)	0.525
Systolic BP, mmHg (mean ± SD)	153.98 ± 21.07	156.72 ± 20.63	150.30 ± 21.23	0.053
Diastolic BP, mmHg (mean ± SD)	87.93 ± 13.27	88.49 ± 13.87	87.17 ± 12.48	0.531
MAP, mmHg (mean ± SD)	109.95 ± 14.43	111.23 ± 14.61	108.21 ± 14.11	0.186
Hypertension, n (%)	137 (83.54)	84 (89.36)	53 (75.71)	0.020
Coronary heart disease, n (%)	8 (4.88)	6 (6.38)	2 (2.86)	0.468
Diabetic retinopathy, n (%)	90 (54.88)	60 (63.83)	30 (42.86)	0.008
Duration of diabetes, years (median, IQR)	8.00 (4.00, 10.00)	10.00 (5.00, 11.00)	6.00 (3.00, 10.00)	0.020
Laboratory				
White blood cell count, 10^9^/L (median, IQR)	6.53 (5.34, 8.20)	6.69 (5.86, 8.45)	6.17 (5.12, 7.47)	0.010
Hemoglobin, g/L (mean ± SD)	113.85 ± 22.08	110.95 ± 20.96	117.76 ± 23.07	0.050
Platelet counts, 10^9^/L (median, IQR)	233.00 (192.75, 281.00)	239.50 (198.00, 285.25)	222.50 (174.50, 264.50)	0.170
Serum uric acid, μmol/L (median, IQR)	370.00 (325.00, 445.25)	382.00 (329.00, 462.50)	358.10 (319.22, 427.05)	0.145
Serum creatinine, μmol/L (median, IQR)	114.50 (83.75, 163.75)	147.00 (101.47, 209.02)	88.40 (69.15, 121.72)	<0.001
eGFR, ml/min/1.73m^2^ (median, IQR)	45.62 (30.75, 68.44)	34.40 (21.83, 53.90)	64.82 (43.30, 86.95)	<0.001
Serum albumin, g/L (mean ± SD)	29.87 ± 7.53	30.04 ± 7.11	29.65 ± 8.10	0.746
Triglycerides, mmol/L (median, IQR)	2.01 (1.33, 3.05)	2.04 (1.39, 3.08)	1.96 (1.27, 3.03)	0.467
Total cholesterol, mmol/L (median, IQR)	5.85 (4.49, 7.52)	5.95 (4.69, 7.52)	5.49 (3.93, 7.46)	0.143
LDL-C, mmol/L (median, IQR)	3.38 (2.42, 4.84)	3.51 (2.46, 4.64)	3.02 (2.27, 4.89)	0.429
HbA1c, % (median, IQR)	7.20 (6.30, 8.50)	7.15 (6.12, 8.07)	7.25 (6.50, 8.60)	0.075
Immunoglobulin G, g/L (median, IQR)	7.34 (6.08, 10.30)	7.48 (6.16, 10.14)	7.29 (5.89, 10.47)	0.807
Fasting blood glucose, mmol/L (median, IQR)	6.15 (4.95, 7.88)	5.96 (4.98, 7.69)	6.15 (4.94, 8.03)	0.503
Hematuria, n (%)	114 (69.51)	65 (69.15)	49 (70.00)	0.907
Proteinuria (g/day) (median, IQR)	5.50 (2.92, 7.32)	6.05 (3.64, 8.93)	4.39 (1.95, 6.46)	0.001
Arteriolar hyalinosis, n (%)	154 (93.90)	89 (94.68)	65 (92.86)	0.629
Oral antidiabetic drugs, n (%)	141 (85.98)	82 (87.23)	59 (84.29)	0.591
Insulin, n (%)	45 (27.44)	33 (35.11)	12 (17.14)	0.011
RAAS inhibitors, n (%)	108 (65.85)	58 (61.70)	50 (71.43)	0.194
SGLT2 inhibitors, n (%)	56 (34.15)	27 (28.72)	29 (41.43)	0.090

Data presented as mean ± standard deviation (SD) or median (IQR) or percentage.

eGFR, estimated glomerular filtration rate; IFTA, interstitial fibrosis and tubular atrophy; LDL-C, Low-density lipoprotein cholesterol; MAP, mean arterial pressure; HbA1c, glycated hemoglobin A1c.

### 3.2 Development of model

**Figure 1:**
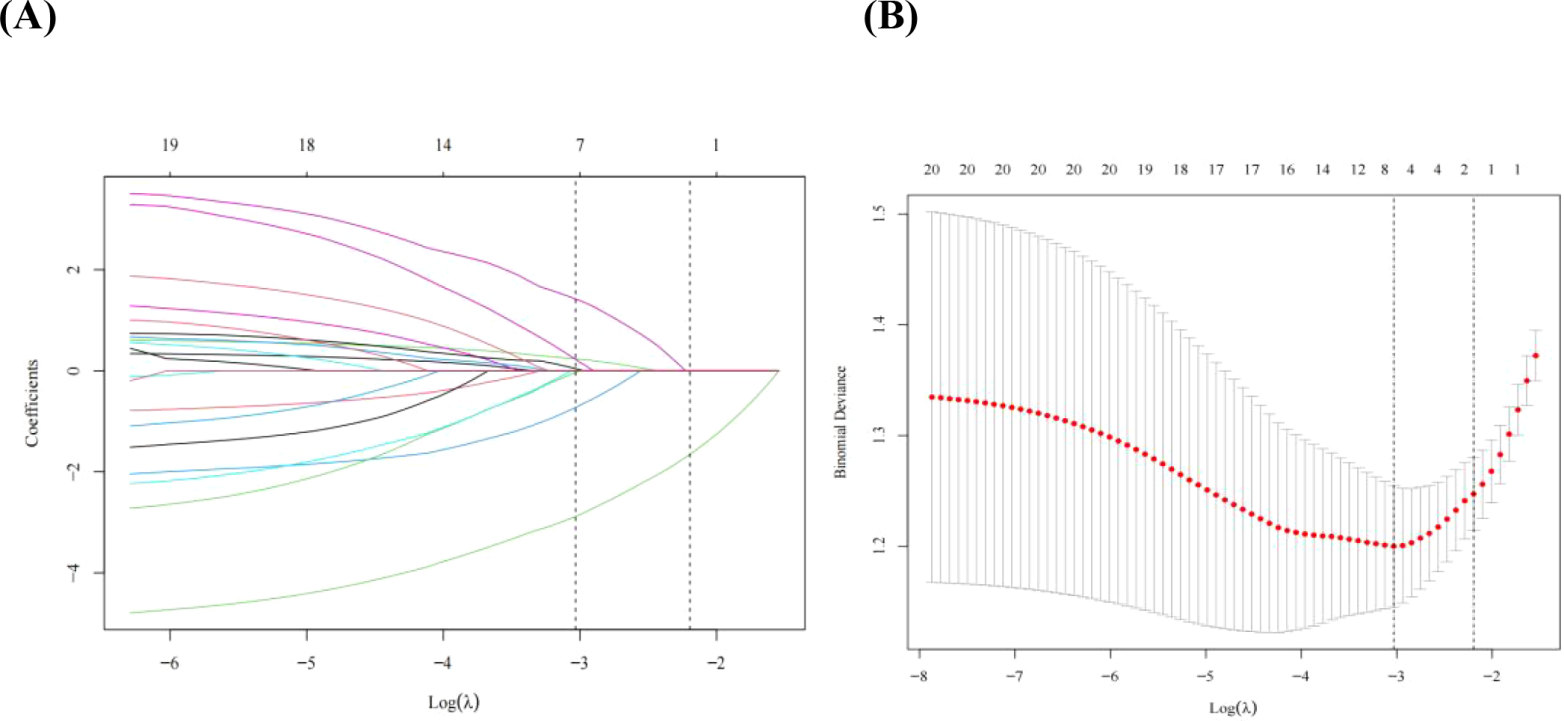
Demographic and clinical feature selection using the LASSO binary logistic regression model. **(A)** Optimal parameter (lambda) selection in the LASSO model used 10-fold cross-validation via minimum criteria. The partial likelihood deviance (binomial deviance) curve was plotted versus log (lambda). Dotted vertical lines were drawn at the optimal values by using the minimum criteria and the 1 SE of the minimum criteria (the 1-SE criteria). **(B)** LASSO coefficient profiles of the 20 features. A coefficient profile plot was produced against the log (lambda) sequence. Vertical line was drawn at the value selected using 10-fold cross-validation, where optimal lambda resulted in seven features with nonzero coefficients. LASSO, least absolute shrinkage and selection operator; SE, standard error.

Among the demographic and clinical variables, 20 candidate features were entered into the LASSO model, and seven predictors with nonzero coefficients were retained in the training cohort (**Figures 1A, B**). The retained predictors were diabetic retinopathy, age, proteinuria, eGFR, triglycerides, duration of diabetes, and hemoglobin, which were subsequently used to develop the machine learning models.

### 3.3 Evaluation of model

**Figure 2:**
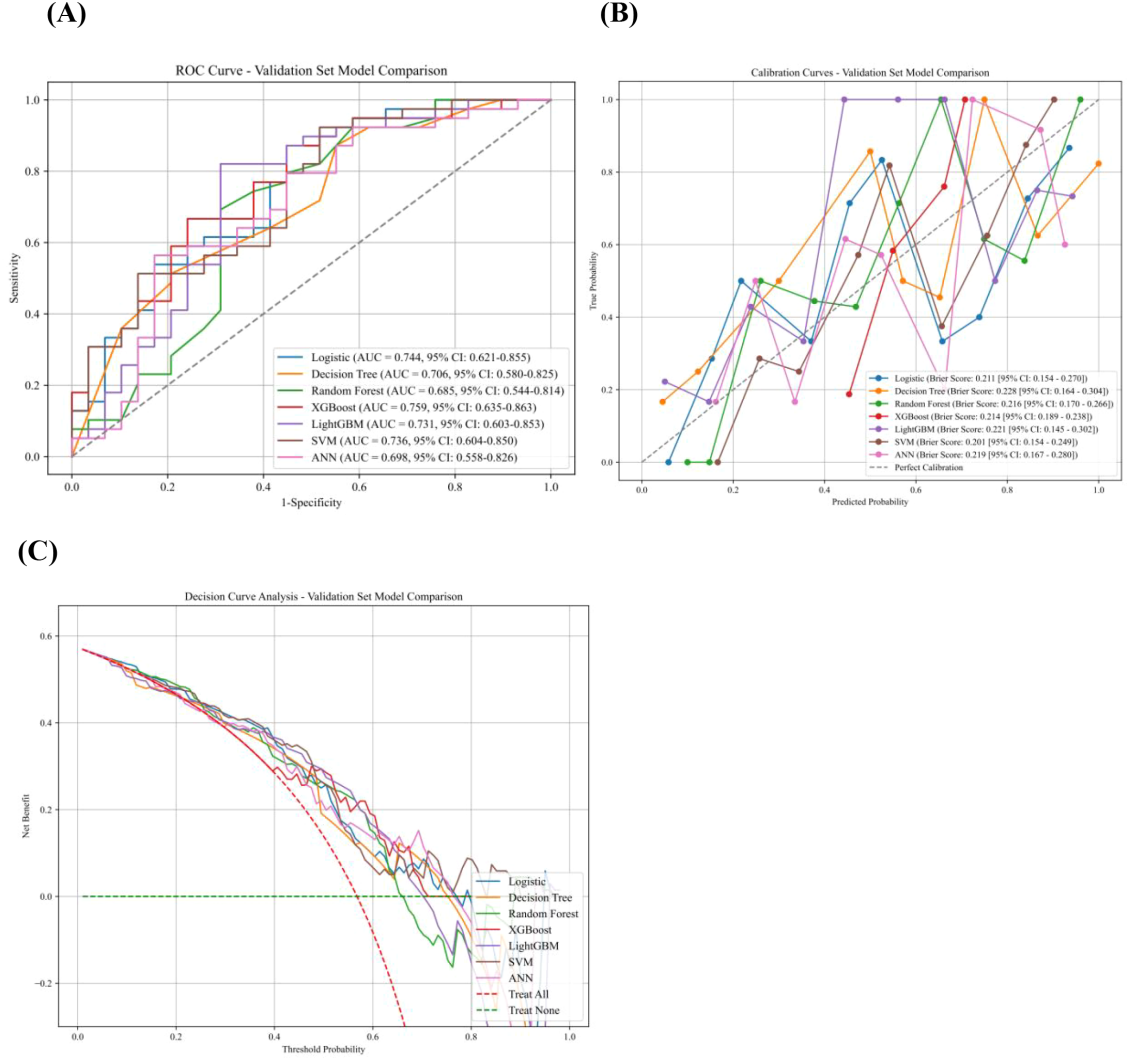
Machine learning-based models used to predict IFTA in patients with DN. **(A)** ROC curves for the machine learning-based models used to predict IFTA. **(B)** Calibration curves with corresponding Brier scores of the machine learning-based models. **(C)** DCA of the machine learning-based models. ROC, receiver operating characteristic; DCA, Decision curve analysis.

Seven machine learning models were developed to predict the occurrence of IFTA in patients with DN. Their performance is shown in **Figure 2**, including ROC curves (**Figure 2A**), calibration curves with Brier scores (**Figure 2B**), and decision curve analysis (**Figure 2C**). Among the evaluated models, XGBoost was selected as the primary model because it achieved the highest AUC in the validation set, although other models such as LightGBM showed a more balanced sensitivity–specificity trade-off. Specifically, XGBoost yielded an AUC of 0.759 (95% CI: 0.635–0.863), an accuracy of 72.1%, sensitivity of 92.3%, precision of 69.2%, specificity of 44.8%, and an F1 score of 79.1% in the validation cohort. The Brier score of the XGBoost model was 0.214 (95% CI: 0.189–0.238), suggesting reasonable calibration. These findings indicate moderate discriminative performance with a sensitivity-oriented classification profile, in which most IFTA-positive cases were detected but at the cost of a relatively high false positive rate. Therefore, although XGBoost showed the highest AUC among the evaluated models, its limited specificity suggests that the current model may be more suitable for exploratory risk screening than for standalone confirmatory clinical use (**Table 3**).

**Table 3:** Performance of seven machine learning-based models for predicting IFTA in the validation set.

Model	AUC	Accuracy	Precision	Sensitivity	Specificity	F1 score	PPV	NPV
LR	0.744	0.721	0.700	0.897	0.483	0.787	0.700	0.778
DT	0.706	0.691	0.667	0.923	0.379	0.774	0.667	0.786
RF	0.685	0.676	0.698	0.769	0.552	0.732	0.698	0.640
XGBoost	0.759	0.721	0.692	0.923	0.448	0.791	0.692	0.813
LightGBM	0.731	0.750	0.775	0.795	0.690	0.785	0.775	0.714
SVM	0.736	0.735	0.706	0.923	0.483	0.800	0.706	0.824
ANN	0.698	0.691	0.673	0.897	0.414	0.769	0.673	0.750

AUC, area under the receiver operating characteristic curve; ANN, artificial neural network; DT, decision tree; GBM, gradient boosting machine; LR, logistic regression; NPV, negative predictive value; PPV, positive predictive value; RF, random forest; SVM, support vector machine; XGBoost, extreme gradient boosting.

### 3.4 Model interpretation

**Figure 3:**
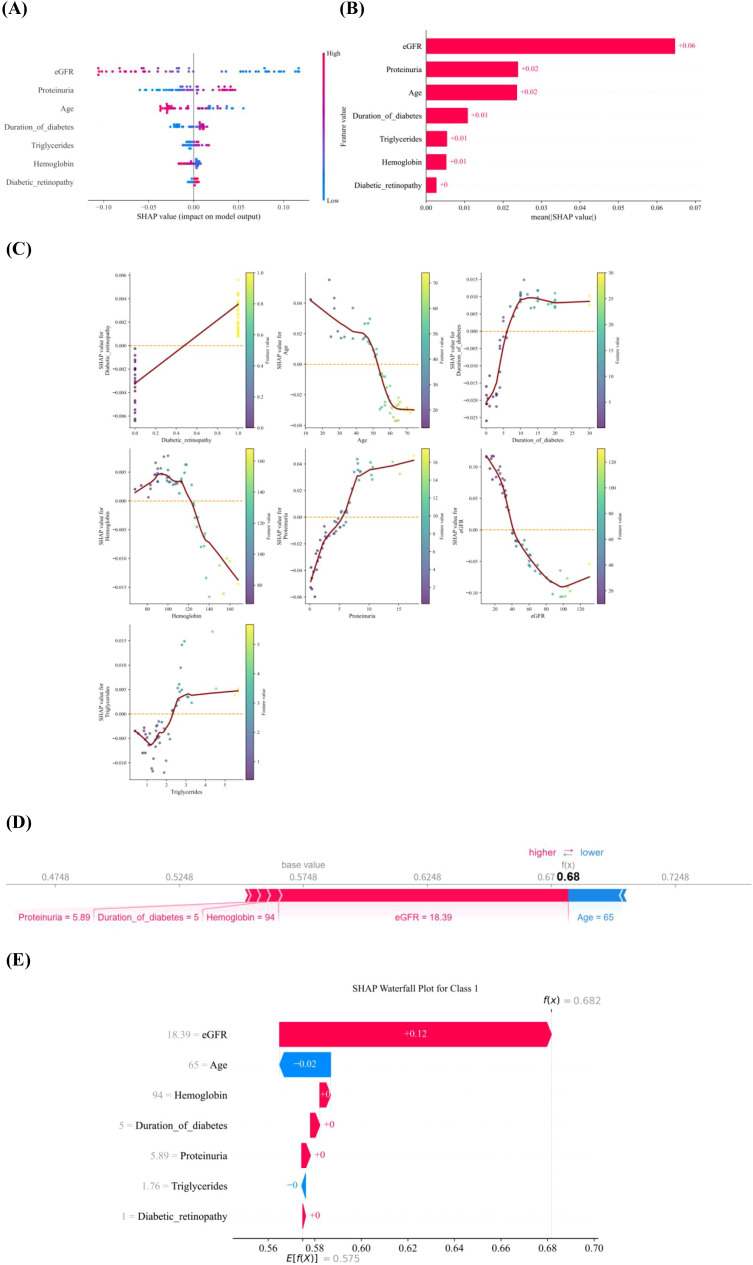
SHAP-based interpretation of the XGBoost model. **(A)** SHAP summary plot showing the distribution of feature effects on model output across all patients. Each dot represents one patient. The x-axis indicates the SHAP value, where positive values increase the predicted probability of IFTA and negative values decrease it. Dot color represents the feature value (red, higher value; blue, lower value). **(B)** Mean absolute SHAP values ranking the overall importance of each predictor in the XGBoost model. **(C)** SHAP dependence plots for the seven most important predictors, illustrating how changes in each feature value relate to its contribution to the predicted risk of IFTA. **(D)** SHAP force plot for a representative individual case, showing how specific features increase or decrease the predicted probability relative to the base value. **(E)** Waterfall plot for the same representative case, illustrating the cumulative contribution of each feature to the final prediction. SHAP, Shapley additive explanations.

The SHAP analysis provided deeper insights into the predictive mechanism of the XGBoost model. As shown in **Figure 3A**, a SHAP summary plot was used to display overall feature importance, while **Figure 3B** presented a bar chart ranking predictors by their mean absolute SHAP values. Furthermore, SHAP dependence plots were applied to visualize the associations between key risk factors and outcomes. The seven most influential features (**Figure 3C**) indicated that higher levels of diabetic retinopathy, proteinuria, triglycerides, and longer diabetes duration, along with lower age, eGFR, and hemoglobin, were strongly linked to an increased risk of IFTA. The dependence plots also illustrated how different ranges of each variable were associated with higher or lower predicted IFTA risk.

The force plot provided personalized feature attribution for one representative example and illustrated how SHAP could be used to explain individual model predictions, as shown in **Figure 3D**. Starting from the base value, defined as the mean prediction across the dataset, each predictor either raised or reduced the probability of IFTA depending on its level, with the arrow length reflecting the magnitude of the SHAP value. **Figure 3E** was the waterfall plot of this individual sample. The contribution of each feature was displayed in red or blue. Red color represented the positive impact on IFTA. The blue color represented negative impact on IFTA. For example, in **Figure 3E**, the eGFR value was 18.39 ml/min/1.73 m², and its SHAP value was 0.682, indicating a substantial positive contribution to the predicted risk of IFTA. The eGFR was in red color and meant a positive impact on prediction of IFTA. This process yielded the final predicted risk for a specific patient, thereby offering an intuitive interpretation of individual model predictions.

## 4 Discussion

In this study, we developed and validated several machine learning–based models to predict the presence of IFTA in patients with biopsy-confirmed diabetic nephropathy. Among the evaluated algorithms, XGBoost achieved the highest AUC in the validation cohort and was therefore selected as the primary model, although other models such as LightGBM showed a more balanced sensitivity–specificity trade-off. However, its predictive ability should be interpreted cautiously, as the model demonstrated moderate discrimination and relatively low specificity despite high sensitivity. Therefore, our findings are best viewed as a proof-of-concept that routinely available clinical variables may provide useful, non-invasive information about underlying tubulointerstitial injury, rather than as evidence for immediate standalone clinical deployment. Overall, the present findings should be interpreted as exploratory and hypothesis-generating rather than as evidence for immediate clinical application.

A key finding of our study is that a limited set of clinically accessible variables was sufficient to achieve meaningful predictive performance. Given the exploratory nature of this retrospective study and the limited sample size, we used LASSO-based feature selection to reduce the number of candidate predictors and minimize the risk of overfitting before model construction. Diabetic retinopathy, proteinuria, eGFR, triglyceride levels, duration of diabetes, hemoglobin concentration, and age emerged as the most informative predictors. These factors reflect complementary dimensions of disease burden, including microvascular injury, metabolic disturbance, and chronic renal dysfunction, underscoring the multifactorial nature of tubulointerstitial damage in diabetic nephropathy.

Proteinuria and eGFR were among the strongest contributors to model predictions, which is consistent with their established roles as markers of renal injury and progression. Previous studies have shown that severe IFTA is closely associated with accelerated decline in renal function and poor renal outcomes in patients with diabetic nephropathy (13, 14). However, functional indicators alone do not fully capture the extent of structural damage. Patients with comparable levels of proteinuria or eGFR may exhibit markedly different degrees of interstitial fibrosis on histopathological examination, highlighting the intrinsic heterogeneity of diabetic nephropathy (13). Our findings support the need for integrated risk assessment strategies that extend beyond single clinical parameters. An additional point that merits emphasis is the relationship between the present model and existing clinical assessment approaches. In routine practice, clinicians already rely on conventional indicators such as proteinuria, eGFR, and diabetic retinopathy to estimate disease severity in diabetic nephropathy. The potential advantage of the current model is not that it replaces these markers, but that it integrates several routinely available variables into a single, interpretable framework for estimating the likelihood of underlying IFTA. This multivariable approach may better reflect the heterogeneous and multidimensional nature of tubulointerstitial injury than any single indicator alone. However, because we did not directly compare the model with established clinical scores, baseline clinical models, or individual biomarkers, the present study cannot prove incremental clinical value. Future studies should formally evaluate whether the proposed model provides added predictive utility beyond conventional clinical assessment.

Hemoglobin level emerged as an important predictor in the present analysis. Anemia is a common complication of chronic kidney disease and is frequently observed in patients with advanced diabetic nephropathy. Reduced hemoglobin levels may reflect impaired erythropoietin production, chronic inflammation, and progressive tubulointerstitial injury (15). Previous studies have demonstrated that lower hemoglobin levels are associated with faster renal function decline and worse prognosis in diabetic nephropathy, supporting its role as an indicator of disease severity rather than an isolated laboratory abnormality (13, 15).

Diabetic retinopathy was also strongly associated with IFTA in our model. As a manifestation of systemic microvascular injury, diabetic retinopathy has been consistently linked to more severe renal histopathological changes, including mesangial expansion, glomerulosclerosis, and tubulointerstitial fibrosis (16). This association reinforces the concept that microvascular complications in diabetes often progress in parallel and that retinal findings may provide indirect insight into renal structural damage.

Metabolic factors contributed meaningfully to the prediction of IFTA. Elevated triglyceride levels and longer duration of diabetes were associated with higher predicted risk, suggesting that prolonged metabolic dysregulation may be associated with chronic renal remodeling, although this interpretation should be viewed cautiously given the observational design and the possibility of residual confounding. Experimental and clinical studies have shown that altered lipid metabolism in renal tubular cells can promote lipotoxicity, mitochondrial dysfunction, and fibrotic signaling pathways, thereby contributing to tubulointerstitial fibrosis (17, 18). These observations emphasize the importance of comprehensive metabolic management in patients with diabetic nephropathy. Emerging evidence also suggests that gut microbiota-related metabolic reprogramming may contribute to the development of metabolic disorders and renal fibrosis. Alterations in intestinal microbiota can influence lipid metabolism, inflammatory pathways, and the production of circulating metabolites such as short-chain fatty acids and trimethylamine N-oxide, which have been implicated in the progression of tubulointerstitial injury (19). These findings suggest that microbiota-related factors may represent an additional dimension for understanding disease heterogeneity and may provide potential targets for future predictive modeling in diabetic nephropathy. Future studies incorporating microbiota-related features may further enhance the predictive performance and biological interpretability of such models.

Interestingly, younger age was associated with a higher predicted probability of IFTA in our cohort. This finding should be interpreted cautiously. Although aging is generally associated with progression of chronic kidney disease, biopsy-based studies have reported similar observations, which may reflect selection bias, as younger patients with atypical presentations or rapid disease progression are more likely to undergo renal biopsy (13). Therefore, this association may reflect characteristics of the biopsy-selected cohort rather than a direct biological relationship.

Beyond predictive performance, interpretability is essential for clinical adoption of machine learning models. In this study, SHAP analysis was used to provide both global and individual-level explanations of model predictions. These techniques allow visualization of how specific clinical features influence predicted risk, enhancing transparency and clinical interpretability (11). By linking model outputs to familiar clinical variables, interpretability tools help bridge the gap between advanced analytics and practical decision-making. Despite additional hyperparameter adjustment, the XGBoost model retained high sensitivity but specificity remained limited, resulting in a relatively high false positive rate in the validation cohort. Several factors may contribute to this finding. First, the study population was derived from a biopsy-based cohort, which may represent a selected group with higher disease severity or atypical clinical presentations, potentially reducing the model’s ability to distinguish milder or non-IFTA cases. Second, the modest sample size may have limited the model’s ability to learn stable decision boundaries, particularly for correctly identifying negative cases. Third, the model was based on routinely available clinical variables and did not incorporate more specific fibrosis-related biomarkers or imaging features that might improve discrimination. Finally, the chosen classification threshold and optimization strategy favored sensitivity, which inherently reduced specificity. From a clinical perspective, this performance pattern suggests that the model may be more useful for exploratory risk screening than for confidently excluding IFTA or supporting standalone clinical decision-making, because false positive predictions could lead to overestimation of risk and possibly unnecessary concern or overtreatment if used in isolation. In clinical practice, such a sensitivity-oriented model may be more appropriate in contexts where missing high-risk patients is undesirable; however, its use would require careful integration with clinical judgment to avoid overestimation of risk. Future studies should explore threshold optimization, cost-sensitive learning, incorporation of additional biomarkers, genetic factors, and imaging features, as well as external validation, to further improve predictive performance and robustness. The integration of more comprehensive multimodal data may also help improve the biological interpretability of model predictions.

An important consideration is why we focused on predicting IFTA rather than direct clinical outcomes. We agree that prediction of renal endpoints such as sustained eGFR decline, kidney failure, or end-stage kidney disease would be highly relevant. Nevertheless, IFTA represents a prognostically meaningful histopathological feature that reflects chronic tubulointerstitial injury and is closely linked to disease severity in diabetic nephropathy. From a clinical perspective, early identification of patients who may be at higher risk of significant tubulointerstitial damage could support closer monitoring and more individualized evaluation of renoprotective strategies, including RAAS blockade and sodium-glucose cotransporter 2 inhibitors, although the clinical utility of this approach remains uncertain and requires further validation (5). Because renal biopsy is invasive and not routinely repeated, a non-invasive framework capable of estimating the likelihood of significant IFTA may still have clinical value, particularly for pathology-oriented risk stratification. Future studies with longitudinal follow-up should determine whether such models are associated with clinically meaningful renal outcomes, including sustained eGFR decline, end-stage kidney disease, and mortality, and whether they provide additional prognostic value beyond conventional clinical indicators. However, we acknowledge that the present study did not evaluate the association between model predictions and longitudinal clinical outcomes. Therefore, whether this approach provides prognostic value beyond estimating pathological injury remains uncertain.

Several limitations should be acknowledged. First, the most important limitation of this study is that it was a retrospective single-center analysis based on a biopsy-selected cohort, which introduces selection bias and substantially limits generalizability. Future studies should include larger multicenter cohorts from diverse geographic regions and healthcare institutions to better evaluate the generalizability and applicability of the model. Second, the sample size was modest, and no formal *a priori* sample size calculation was performed. Although 94 IFTA events were available in the training cohort and the final model included 7 predictors after LASSO-based feature selection, these numbers should be interpreted cautiously because conventional events-per-predictor considerations do not directly apply to machine learning models and do not exclude the possibility of overfitting. Therefore, the present study should be regarded as exploratory and proof-of-concept in nature. In addition, no independent external validation cohort was available, which further limits assessment of model stability and generalizability across different populations and clinical settings. Third, although all biopsy specimens were independently reviewed by two experienced renal pathologists and discrepancies were resolved by consensus, formal inter-observer agreement statistics were not available. Fourth, the best-performing model showed relatively low specificity, resulting in a high false positive rate and limited ability to correctly identify patients without IFTA. This reduces its potential utility as a standalone decision-support tool and suggests that its current value lies mainly in exploratory risk stratification rather than definitive classification. In addition, confidence intervals were reported only for AUC, whereas confidence intervals for other performance metrics were not provided. Although Brier scores were presented in the calibration plots, more comprehensive reporting of calibration statistics and internal validation variability would strengthen the robustness of performance assessment. Fifth, the model was based on routinely available clinical variables and did not incorporate additional fibrosis-related biomarkers, genetic factors, or imaging-derived indicators, all of which might have improved predictive performance and biological interpretability. We also did not perform direct comparisons with existing clinical scoring approaches, baseline clinical models, or single biomarkers; therefore, the incremental value of the proposed model over conventional clinical assessment remains uncertain. Sixth, model performance was evaluated using a single random 7:3 training-validation split, and repeated random splits or bootstrap resampling were not performed. Therefore, the stability and variability of the reported performance estimates could not be fully assessed. Finally, we focused on the prediction of pathological IFTA rather than longitudinal clinical outcomes. We did not assess the association between model predictions and patient prognosis, such as renal function decline, end-stage kidney disease, or mortality. Therefore, the prognostic value and clinical impact of the model remain uncertain and require further investigation in longitudinal studies.

## 5 Conclusion

In conclusion, this study suggests that machine learning models, particularly XGBoost, may provide moderate non-invasive predictive value for estimating the likelihood of IFTA in patients with biopsy-confirmed diabetic nephropathy using routinely available clinical data. However, given the sensitivity-oriented performance profile, limited specificity, retrospective single-center design, biopsy-based participant selection, and lack of external validation, the present findings should be considered exploratory. The relatively high false positive rate may lead to overestimation of IFTA risk and potential overdiagnosis or overtreatment if used in isolation. Therefore, the current model should not be regarded as a standalone clinical decision-making tool. In addition, because the relationship between model predictions and clinical outcomes was not evaluated, the prognostic relevance of this approach remains to be established. Further studies, particularly prospective cohort studies with longitudinal follow-up, are needed to optimize model performance, validate it in independent cohorts, and determine whether this approach has practical value for predicting clinically relevant renal outcomes in real-world settings.

## Data Availability

The raw data supporting the conclusions of this article will be made available by the authors, without undue reservation.
